# Oral Multienzyme Supplementation Alters Postprandial Plasma Nutrient Concentrations after a Mixed Meal in Healthy Middle-Aged and Older Adults: A Randomized, Double-Blind, Placebo-Controlled, Crossover Trial

**DOI:** 10.1016/j.tjnut.2026.101400

**Published:** 2026-02-07

**Authors:** Max T Deutz, Andrew T Askow, Sean M Garvey, David A Alvarado, Takeshi M Barnes, Žan Zupančič, Alexander V Ulanov, Jared W Willard, Hannah D Holscher, Brett R Loman, Nicholas A Burd

**Affiliations:** 1 Division of Nutritional Sciences, University of Illinois Urbana-Champaign, Urbana, IL; 2 Department of Health and Kinesiology, University of Illinois Urbana-Champaign, Urbana, IL; 3 Department of Research and Development, BIO-CAT, Inc., Troy, VA; 4 SAPIOME LLC, Jericho, VT; 5 Roy J. Carver Biotechnology Center, University of Illinois Urbana-Champaign, Urbana, IL; 6 Christie Clinic, Orthopedics & Sports Medicine, Champaign, IL; 7 Department of Food Science and Human Nutrition, University of Illinois Urbana-Champaign, Urbana, IL; 8 Department of Animal Sciences, University of Illinois Urbana-Champaign, Urbana, IL

**Keywords:** amino acids, enzymes, free fatty acids, glucose, nutrition, aging

## Abstract

**Background:**

Age-related decline in digestive function increases malnutrition risk. Supplementing meals with digestive enzymes may improve macronutrient digestion and bioavailability in adults reaching older ages.

**Objectives:**

To assess postprandial plasma nutrient concentrations after co-ingestion of a mixed meal and a mixture of 6 enzyme preparations (ENZ), including proteases, lipase, amylase, and glucoamylase.

**Methods:**

Thirty middle-aged and older adults (56 ± 11 y; 18 females, 12 males) ingested chicken, peas, potatoes, and butter (435 kcal; 34 g protein, 51 g carbohydrate, 11 g fat) with either ENZ or placebo (PLA) in a randomized crossover fashion. Blood samples were collected at baseline and throughout a 0–5 h postprandial period for measurement of plasma amino acid, insulin, glucose, and nonesterified fatty acid (NEFA) concentrations. Clustering of postprandial amino acid responses was conducted in MFuzz, and logistic regression for response groups was conducted in JMP 18.2.0 (JMP Statistical Discovery LLC).

**Results:**

Plasma amino acid concentrations were not statistically different between treatments (PLA compared with ENZ) over the postprandial period (all *P >* 0.05). Leucine time to maximum concentration was significantly faster (*P =* 0.047) with ENZ (121.2 ± 55.9 min) compared with PLA (141.0 ± 49.2 min). Postprandial plasma glucose concentrations (*P* = 0.04) and total NEFA (*P* = 0.001) were higher with ENZ compared with PLA. Three distinct response patterns (clusters) were detected within and across all postprandial amino acid categories. Differences in habitual macronutrient intake and interactions between sex, lean mass, and BMI distinguished participants with an earlier time to maximum postprandial leucine concentration when consuming ENZ compared with PLA from those with stable responses.

**Conclusions:**

Multienzyme supplementation improved macronutrient digestion of a mixed meal in middle-aged and older adults. For plasma amino acids, this benefit was most pronounced in adults with lower BMI and higher lean mass, and the effect was sex-dependent.

This study was registered at clinicaltrials.gov as NCT05211440.

## Introduction

With projections of exponentially rapid growth among older populations, age-associated deleterious health effects are critically important to both individual and public health [1]. Although the progressive age-related loss of skeletal muscle mass, strength, and cognitive function are well documented, the impact of aging on the gastrointestinal system remains less clear [2,3]. As early as the fifth decade of life, gastrointestinal function has been shown to worsen, as evidenced by reduced gastric acid and pepsin output, delayed gastric emptying, slower buffering of postprandial gastric pH, decreased pancreatic enzyme secretion, and lower bicarbonate output [[4], [5], [6], [7], [8], [9], [10], [11], [12], [13], [14], [15], [16], [17], [18]]. Notably, this reduction in bicarbonate can alter duodenal pH and pancreatic enzyme activities [7,[14], [15], [16], [17], [18]]. In addition to gastric and pancreatic changes, intestinal bile concentrations can also decline with advancing age [19,20]. Collectively, these age-related gastrointestinal changes have been termed digestive senescence [21]. Aging is also associated with reduced appetite and dietary intake, increasing the risk of malnutrition, chronic weight loss, and muscle wasting [22]. Although established interventions like nutritional assistance (e.g., meal preparation and counseling) and increased physical activity can help protect against such declines, emerging secondary strategies such as digestive enzyme supplementation are also being explored to support the health of adults reaching older ages [[23], [24], [25], [26], [27]].

Enzymes manufactured via microbial fermentation, or microbial enzymes, have shown broad pH tolerance and demonstrated efficacious hydrolytic action toward dietary substrates within in vitro gastrointestinal digestion simulations and mouse models [21,[28], [29], [30], [31], [32]]. Human studies using such enzymes have primarily focused on mitigating pre-existing gastrointestinal symptoms, including functional dyspepsia or gluten sensitivity [[33], [34], [35], [36], [37]]. Clinical trials have also explored the effect of enzymes on enhancing protein digestion and absorption kinetics by examining postprandial plasma amino acid concentrations in healthy young adults [[38], [39], [40], [41], [42]]. A research gap exists regarding digestive senescence, especially considering assessment of mixed meal dietary patterns is more ecologically valid than isolated protein sources. Accordingly, studies are warranted to investigate the effects of enzyme supplementation on postprandial plasma nutrient concentrations after mixed meal ingestion in middle-aged and older adults to clarify its utility in supporting healthy aging.

The primary aim of this clinical trial was to assess whether enzyme supplementation acutely modulates postprandial plasma amino acid concentrations after the ingestion of a standardized mixed meal in healthy middle-aged and older adults. We hypothesized that supplementation with a mixture of 6 enzyme preparations (ENZ) would complement innate endogenous human digestive enzymes to improve the digestion and absorption of food, thereby significantly elevating plasma amino acid concentrations when compared with placebo (PLA) in a crossover design. Secondary endpoints after consumption of the mixed meal included postprandial plasma insulin, glucose, and total nonesterified fatty acids (NEFA) concentrations and subjective appetite sensations. Subjective gastrointestinal symptoms, bowel habits, and sleep quality were also assessed across 3 wk of twice daily ENZ supplementation. These aims were ultimately directed at an overarching objective of defining how enzyme supplementation impacts aspects of nutrient availability [ie, incremental AUC (iAUC), time to maximum concentration (*T*_max_), or maximum concentration (*C*_max_)] to support healthy aging.

## Methods

### Study design and ethical approval

A randomized, double-blind, placebo-controlled, crossover study of healthy middle-aged and older adults was conducted from January 2022 to August 2023 at a single research site (University of Illinois Urbana-Champaign, Urbana, IL, United States) to test the effects of ENZ on postprandial changes in nutrient concentrations after consuming a standardized mixed meal. The clinical trial consisted of an online screening visit and 5 onsite visits (visits 1–5) that included a secondary screening, 2 investigational product distributions (at the beginning of each treatment period), and 2 acute meal treatment tests at the end of each 3-wk supplementation period. All visits conformed to standards for the use of human participants in research as outlined in the Helsinki Declaration, and all study procedures were reviewed and approved by the Institutional Review Board (IRB) at the University of Illinois Urbana-Champaign (IRB number 22237). The protocol was amended on 15 September, 2022, to expand the age eligibility range from 50–74 y to 40–75 y (inclusive). The study was registered at clinicaltrials.gov (NCT05211440).

### Investigational products

The mixture of 6 nongenetically engineered supplemental microbial enzyme preparations (trade name: OPTIZIOME Macro Digest, lot nos. W0023521 and W0032092; BIO-CAT, Inc.), known herein as “ENZ,” has previously been described and is also summarized in Supplemental Table 1 [21,28]. Briefly, ENZ contains 3 protease preparations manufactured by traditional Japanese kōji fermentation of wild-type *Aspergillus* species, a lipase from *C. cylindracea*, amylase from *A. oryzae*, and glucoamylase from *A. niger*. ENZ is formulated into size 1 cellulose capsules that also contain the following excipients and flow agents: magnesium stearate (5 mg), silicon dioxide (1 mg), and additional maltodextrin (10 mg; Cargill Inc.). PLA contains maltodextrin with the same excipients and flow agents.

### Participants

Individuals were recruited for participation using posted community flyers, in-person tabling events, and University-sanctioned email bulletins. A total of 112 individuals were assessed for eligibility. Inclusion criteria sought generally healthy adult males and females aged between 40‒75 y with BMI 18‒29.99 kg/m^2^ and fasting blood glucose level ≤100 mg/dL. Exclusionary medications and supplements were clearly outlined and, notably for the latter, included recent and/or regular consumption of either enzymatic or pre/probiotic supplements. Accordingly, 30 middle-aged and older adults (18 females, 12 males; 25 ± 2.2 kg/m^2^; 56 ± 11 y) volunteered and were enrolled as participants in this study. Participant characteristics are shown in Table 1. All participants were deemed healthy and physically active based on their responses to both a routine medical screening questionnaire and the International Physical Activity Questionnaire completed during the online screening. Additionally, all participants were informed about the experimental procedures, the purpose of the study, and potential risks before providing informed consent to continue. After completion of the online screening, eligible participants underwent an onsite secondary screening where they reported to the laboratory in the morning after an overnight fast (12 ± 2 h) for measurement of body mass, height, and body composition via dual-energy X-ray absorptiometry (Horizon W; Hologic Inc.). After this onsite secondary screening visit, eligible participants who were willing to continue were randomized for enrollment. A computer-generated list of random numbers was used for participant allocation. The study product was bottled/coded with a random computer-generated number then distributed to blinded researchers via a third party not involved in data collection/analysis. All associated researchers and participants were kept blinded until data collection/analysis concluded.

**TABLE 1:** Baseline characteristics of healthy participants who completed the crossover clinical trial according to the study protocol

Variable	Male (*n* = 10)	Female (*n* = 15)	Total (*n* = 25)
Age (y)	58 ± 13	55 ± 10	56 ± 11
Height (m)	1.78 ± 0.07	1.67 ± 0.06	1.71 ± 0.09
Body mass (kg)	83.1 ± 8.3	69.5 ± 9.3	74.9 ± 11.1
Fat free mass (kg)	58.1 ± 5.6	41.1 ± 4.8	47.9 ± 9.9
Body fat (%)	29.8 ± 6	40.7 ± 3.3	36.3 ± 7
Body mass index (kg⋅m^−2^)	26.1 ± 1.7	24.8 ± 1.8	25.3 ± 1.8
SBP (mmHg)	123.9 ± 7.1	115.1 ± 9.6	118.6 ± 9.6
DBP (mmHg)	75.9 ± 8.4	76.6 ± 7.8	76.3 ± 7.9
Fasting glucose (mmol·L^−1^)	5.00 ± 0.43	4.98 ± 0.38	4.99 ± 0.39

Data are mean ± SD.

Abbreviations: DBP, diastolic blood pressure; SBP, systolic blood pressure.

### Study procedures

**FIGURE 1:**
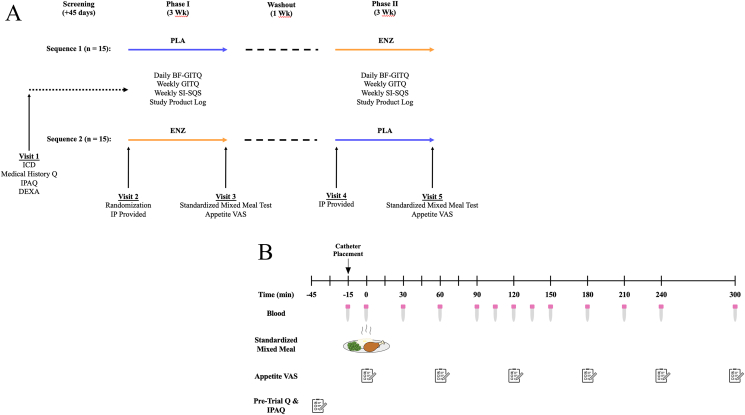
Schematics of the randomized, placebo-controlled, crossover clinical trial design (A) and acute treatment visit protocol including administration of a standardized mixed meal with postprandial blood sampling and subjective appetite questionnaires (B). The standardized mixed meal contained grilled chicken breast, steamed peas, instant potatoes, milk, and butter (435 kcal; 34 g protein; 51 g carbohydrate; 10 g fat). BF-GITQ, Bowel Function and Gastrointestinal Tolerance Factors Questionnaire; DEXA, dual-energy X-ray absorptiometry; ENZ, mixture of 6 microbial enzyme preparations; GITQ, Gastrointestinal Tolerance Questionnaire; ICD, informed consent document; IP, investigational product (PLA or ENZ); IPAQ, International Physical Activity Questionnaire; PLA, placebo; Q, questionnaires; SI-SQS, Single-Item Sleep Quality Scale; VAS, visual analog scale.

An overview of the experimental protocol is shown in Figure 1A. At visit 2, paper questionnaires were provided alongside a bottle of coded investigational product with instructions for dietary supplementation. Participants were directed to consume 1 capsule alongside each of their 2 largest daily meals (self-selected) for 3 wk. Participants recorded the date and time that each capsule was consumed. Participants were also instructed to complete daily and weekly paper questionnaires related gastrointestinal symptoms, bowel habits, and sleep endpoints. Specifically, a Bowel Function and Gastrointestinal Tolerance Factors Questionnaire was completed daily, and the Gastrointestinal Tolerance Questionnaire (GITQ) and Single-Item Sleep Quality Scale (SI-SQS) were completed weekly [[43], [44], [45]]. At visit 2, a standardized meal was also provided for consumption the evening before their first treatment visit. This meal contained ∼25%‒30% of daily energy requirements with energy derived from ∼50% carbohydrate, ∼25% protein, and ∼25% fat. In addition, participants were instructed to maintain the same dietary intake for 72 h prior to each treatment visit, and dietary intake was evaluated using the Automated Self-Administered 24 Hour Dietary Assessment Tool (version 2020; National Cancer Institute). Finally, participants were also instructed to refrain from any strenuous physical exercise or alcohol consumption for 72 h and 48 h, respectively, prior to each treatment visit.

**FIGURE 2:**
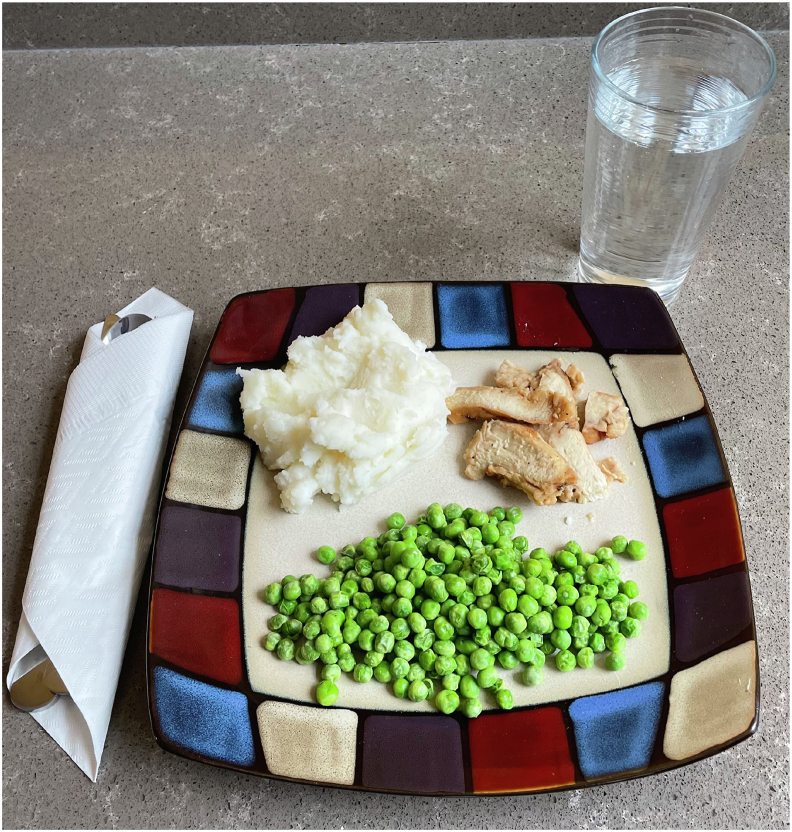
Photograph of the mixed meal consumed by healthy middle-aged and older adults at each of the acute treatment visits in a randomized, placebo-controlled, crossover clinical trial. The standardized meal provided 435 kcal (34 g protein; 51 g carbohydrate; 11 g fat).

On the morning of each of the 2 treatment visits (i.e., visits 3 and 5), participants reported to the laboratory in the morning after an overnight fast (12 ± 2 h). An overview of the acute treatment visit protocol is shown in Figure 1B. After assessment of blood pressure and any potentially reported adverse events, an intravenous catheter was inserted into an antecubital vein, and an arterialized baseline blood sample was collected (*t* = –15 min). Subsequently, participants ingested a standardized mixed meal that consisted of 75 g grilled chicken breast strips (Tyson Grilled & Ready Chicken Breast Strips; Tyson Foods LLC), 200 g instant mashed potatoes (Idahoan Original Mashed Potatoes; Idahoan Foods LLC), 11 g unsalted butter (Great Value; Walmart Apollo LLC), 4 oz milk (2%) (Great Value; Walmart Apollo LLC), and 120 g steamed green peas (Birds Eye Steamfresh Sweet Peas; Pinnacle Foods Group LLC). The standardized mixed meal provided 435 kcal, 34 g protein, 51 g carbohydrate, and 11 g fat and is shown in Figure 2. The investigational product (ENZ or PLA) was consumed with the first 2 to 3 bites of the meal, consistent with instructions outlined during the 3-wk supplementation period. Participants were allowed to drink water ad libitum throughout their first treatment visit, and equal volumes were provided and consumed during the second treatment visit.

Immediately after the standardized mixed meal and investigational product were ingested, a second arterialized blood sample was collected. Additional arterialized blood samples were collected during the postprandial period (*t =* 30, 60, 90, 105, 120, 135, 150, 180, 210, 240, and 300 min) when participants were given the freedom to pass time doing completely sedentary leisurely activities (e.g., reading). Blood samples were immediately centrifuged at 3000 × *g* for 10 min at 4°C, and the plasma was subsequently aliquoted and stored at −80°C. Additionally, during each of these acute treatment visits, measures related to appetite were assessed using a visual analog scale (VAS) questionnaire (validated and adapted from Stubbs et al. [46] and Flint et al. [47]) at baseline (*t* = –15 min), immediately after the standardized mixed meal (*t* = 0 min), and at *t* = 60, 120, 180, 240, and 300 min postprandially. A total of 12 VAS measures were assessed and included questions directly assessing appetite (e.g., “How strong is your feeling of fullness?”) to others that sought out relation via more indirect, yet still related, questions “How strong is your grip?”). Scores for VAS measures were determined via digital sliding scales whereby the slider started halfway between the minimum score [i.e., farthest left; 0 arbitrary units (AU)] and maximum score (i.e., farthest right; 100 AU), and participants moved the slider to the position that most reflected their subjective feelings of each appetite sensation.

After a washout period (≥1 wk and ≤4 wk), participants returned to the laboratory for a second onsite investigation product distribution at visit 4. A subsequent 3-wk supplementation period and acute treatment visit (i.e., visit 5) were completed in an identical fashion to the prior 3-wk supplementation period and acute treatment visit, apart from consuming the alternate investigational product.

Although macronutrients such as protein, fat, and starch are not typically considered major triggers of food sensitivity, it seemed reasonable to assess gastrointestinal tolerance across 3 wk of daily supplementation as multienzyme mixtures including microbial proteases have previously been shown to reduce gut symptomology across 2 wk and 60 d of supplementation in individuals with nonceliac gluten sensitivity and functional dyspepsia, respectively [33,35]. The 1-wk washout period was expected to be sufficient, given the acute mechanism of enzyme action on food substrates without major impacts on the bioaccessibility of nutrients for the gut microbiome.

### Plasma assessments

Plasma amino acid concentrations were determined via a liquid chromatography–tandem mass spectrometry system [LC-MS/MS; Altis Triple Quadrupole LC-MS/MS (equipped with Vanquish HPLC); Thermo Fisher Scientific]; a detailed description of the methods for sample preparation and analysis are provided in the Supplemental Methods. Plasma total NEFA concentrations were determined using a LC-MS/MS system [1290 Infinity II UPLC system (connected to a 9465C Triple Quadrupole MS); Agilent Inc.]; a detailed description of the methods for sample preparation and analysis are provided in the Supplemental Methods. Plasma glucose concentrations were determined using an automated biochemical analyzer (2900 Stat Plus; YSI Life Sciences). Plasma insulin concentrations were determined using commercially available ELISA (catalog number. 80-INSHU-CH01; ALPCO).

### Statistical analyses

#### Sample size calculation

The target enrollment of 30 participants was based on a power analysis for the primary endpoint—change between ENZ and PLA treatments in plasma essential amino acid (EAA) time series and mean incremental AUC (iAUC) after consumption of a mixed meal. Sample size was determined based on a priori power calculation performed using R version 3.6.2 (R Core Team, 2020) for estimating likely differences between mean iAUC for plasma EAA concentrations and utilized prior data comparing the mean total EAA AUC between isolated protein with placebo and isolated protein with probiotic (181,071 compared with 210,115 μmol/L/180 min) to yield a Cohen’s *d* of 0.60 [48]. To detect a medium effect size at 85% power, the analysis indicated a sample of *n* = 27 would be required for a 2-tailed *t*-test between means with alpha at 0.05. Accounting for a 10% dropout rate, a total of 30 participants were recruited for this study.

#### Data calculations

The level of statistical significance was set at *P* < 0.05 for all analyses, and the false discovery rate was applied to exploratory secondary outcomes. All data are presented as mean ± SD, unless otherwise noted. All inferential statistics were analyzed using IBM Statistics for Windows (V29.0.2.0). Data were assessed for normality via visual inspection of normal Q-Q plots and via interpretation of skewness and kurtosis values. Heteroscedasticity was assessed via inspection of a residual plot over the range of values. Homogeneity of variance was assessed using Levene’s test for equality of variances, and sphericity was assessed using Mauchly’s test for sphericity. Bonferroni post hoc correction was applied for calculation of pairwise comparisons when significant main effects or interactions were identified. Time series data (i.e., all plasma amino acid, insulin, glucose, and total NEFA concentrations plus BF-GITFQ, VAS, and SI-SQS measures) were analyzed using linear mixed effects models with time and treatment as fixed factors and participant intercept as a random effect. GITQ measures were analyzed using the McNemar test. Time-independent data [i.e., mean iAUC (as described and adapted from Brouns et al. [49]), *T*_max_, *C*_max_, etc.] were analyzed using linear mixed effects models with treatment as a fixed factor and participant intercept as a random effect. Mean iAUC was calculated within Microsoft Excel. Briefly, baseline analyte concentrations were subtracted from values after *t* = 0 min; subsequently, the weighted average of adjacent time points was calculated and summed before dividing by the total duration (i.e., 300 min). For primary, secondary, and exploratory plasma amino acid endpoints, the mean iAUC for the 0‒5 h postprandial period was calculated. The postprandial window was designated as such, considering the typical time frame of postprandial peak amino acid availability, specifically keeping in mind the use of an older test population and whole food mixed meal test substrate [[50], [51], [52], [53], [54]]. For plasma insulin and glucose, the mean iAUC for the postprandial period was also calculated. Given the small amount of missing data [∼3% for primary endpoints (∼25 missing time points out of 750)] and the ability of these previously outlined mixed models to handle such voids, no imputations were computed for missing data. Cohen’s *d* was calculated as a measure of effect size (0.20 = small effect size; 0.50 = medium effect size; 0.80 = large effect size) of iAUC for the primary EAA endpoint.

#### Postprandial amino acid clustering and modeling

Postprandial concentrations of *1*) EAAs, *2*) leucine, *3*) branched chain amino acids (BCAAs), and *4*) total amino acids (TAAs) were subjected to soft clustering utilizing MFuzz [55]. In this technique, similar response patterns are clustered in a data-driven manner, and the fit (or membership) of each sample is determined as the percentage of all clusters. The appropriate number of clusters to utilize (in all cases, 3) was determined when ≥90% of the samples had a maximum membership ≥40%, and the minimum difference between the 2 highest memberships was ≥10%. Cluster 2 was defined as the “ideal” postprandial amino acid response because it showed a faster *T*_max_ without impacting the duration of elevation, which may be relevant to counteract the compromised protein digestion and absorption kinetics commonly observed with aging [56]. Particularly, this age-related decline limits peak postprandial plasma amino acid concentrations after a meal (particularly leucine), which is likely a contributing factor to the delayed postprandial muscle protein synthetic response and overall anabolic resistance [57,58]. In 14 cases, clustering of a single amino acid group was inconsistent with the other 3 within the given participant and treatment arm. In these and all other cases (where all 4 responses were consistent), the average response (cluster) was assigned. Comparison of PLA and ENZ responses were deemed: *1*) “stable,” when PLA and ENZ responses fit the same cluster; *2*) “improved,” when PLA to ENZ response shifted from cluster 1 to 2 or 3, or cluster 3 to 2; and *3*) “worsened,” when PLA to ENZ response shifted from cluster 2 to 3 or 1, or cluster 3 to 1.

Logistic regression was conducted in JMP (V18.2.0) to detect factors that predict an improved compared with stable response to ENZ. The initial model included all main and 2-way interaction effects of sex, age, BMI, fat mass (as a percentage), and lean mass (in kilograms). The term with the highest *P* value (excluding main effects for which an interaction term remained in the model) was progressively removed from the model until all following criteria were met: *1*) the whole model χ^2^ test *P* < 0.05; *2*) the lack of fit χ^2^ *P* > 0.05; and *3*) parameter estimates were stable.

## Results

### Participants

Of the 30 healthy middle-aged and older adults who were randomized for participation, 29 completed the study. Participant baseline and anthropometric characteristics are shown in Table 1. The 1 participant who withdrew during the first supplementation period cited lack of availability. Four participants failed to comply with either investigational product consumption (defined by >80% and <120% twice daily consumption) or completion of the entire mixed meal during an acute treatment visit. Therefore, the full analysis set comprises the 25 participants who completed the study and adhered to critical study protocols. Investigational product compliance during the 3-wk supplementation periods were 99.5% ± 1.0% for ENZ and 98.2% ± 2.3% for PLA.

### Primary endpoints

#### EAAs

**FIGURE 3:**
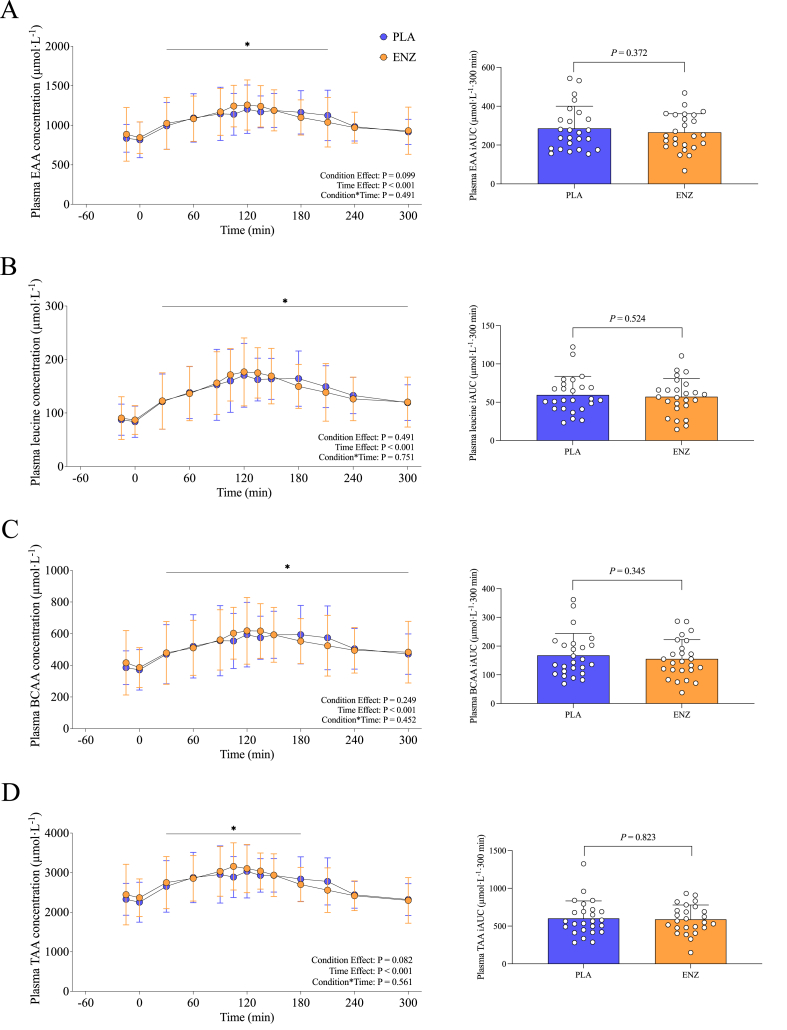
Plasma EAA (A), leucine (B), BCAA (C), and TAA (D) mean postprandial time series concentrations (in micromoles per liter per 300 minutes) of participants who consumed a mixed meal with PLA or ENZ in a crossover clinical trial. Corresponding insets show iAUC (in micromoles per liter per 300 minutes) concentrations. Values are means ± SD. ∗Denotes significant effect of time (*t* = –15; *P* < 0.05). BCAA, branched chain amino acids; EAA, essential amino acids; ENZ, mixture of 6 microbial enzyme preparations; iAUC, incremental AUC; PLA, placebo; TAA, total amino acids.

Plasma EAA concentrations increased, regardless of treatment, during the postprandial period (*P* < 0.001), but no statistical significance was observed for treatment (*P* = 0.099) or treatment × time (*P* = 0.491) (Figure 3A). Plasma EAA iAUC also demonstrated no statistical significance (*P* = 0.372) between PLA (285.8 ± 114.4 μmol/L/300 min) or ENZ (265.7 ± 95.4 μmol/L/300 min) (Figure 3A). Similarly, plasma EAA *T*_max_ (PLA: 136.2 ± 40.8 min, ENZ: 112.2 ± 63.5 min; *P* = 0.089) and *C*_max_ (PLA: 1383.2 ± 330.6 μmol/L/300 min, ENZ: 1412.7 ± 372.9 μmol/L/300 min; *P* = 0.572) failed to reach statistical significance between treatments (Table 2).

**TABLE 2:** Postprandial plasma amino acid concentration incremental AUC (iAUC), maximal concentration (*C*_max_), and time to maximal concentration (*T*_max_) of participants who consumed a mixed meal with a mixture of 6 microbial enzyme preparations (ENZ) or placebo (PLA) in a crossover clinical trial

		ENZ	PLA	*P* value	Cohen’s *d*
Leu	iAUC^1^	57.2 ± 23.7	59.6 ± 24.1	0.524	–0.102
	*C*_max_^2^	204.7 ± 66.2	202.9 ± 62.0	0.813	0.028
	*T*_max_^3^	121.2 ± 55.9	141.0 ± 49.2	0.047	–0.376
BCAA	iAUC^1^	155.6 ± 67.0	167.8 ± 76.4	0.345	–0.170
	*C*_max_^2^	698.8 ± 234.4	691.6 ± 221.6	0.809	0.031
	*T*_max_^3^	124.8 ± 60.3	138.0 ± 39.4	0.299	–0.259
EAA	iAUC^1^	265.7 ± 95.4	285.8 ± 114.4	0.372	–0.191
	*C*_max_^2^	1412.7 ± 372.9	1383.2 ± 330.6	0.572	0.084
	*T*_max_^3^	112.2 ± 63.5	136.2 ± 40.8	0.089	–0.450
TAA	iAUC^1^	589.5 ± 189.5	602.2 ± 231.1	0.823	–0.060
	*C*_max_^2^	3607.8 ± 736.8	3434.0 ± 649.1	0.176	0.250
	*T*_max_^3^	102.0 ± 48.2	122.4 ± 46.4	0.148	–0.431

Data are mean ± SD.

BCAA, branched chain amino acids; EAA, essential amino acids; TAA, total amino acids.

1 Units for iAUC are micromoles per liter per 300 minutes.

2 Units for *C*_max_ are micromoles per liter.

3 Units for *T*_max_ are minutes.

## Secondary endpoints

### Other amino acids

Plasma leucine, BCAA, and TAA concentrations increased after mixed meal ingestion with ENZ and PLA (*P* < 0.001), but no statistical significance was observed for treatment, treatment × time, or mean iAUC (μmol/L/300 min) (all *P* > 0.05) (Figure 3B–D). Likewise, there were no statistically significant differences in leucine, BCAA, and TAA *C*_max_ (μmol/L) (all *P* > 0.05) or BCAA and TAA *T*_max_ (min) between treatments (Table 2). Leucine *T*_max_ was significantly faster (*P* = 0.047) with ENZ (121.2 ± 55.9 min) compared with PLA (141.0 ± 49.2 min) (Table 2).

#### Insulin

**FIGURE 4:**
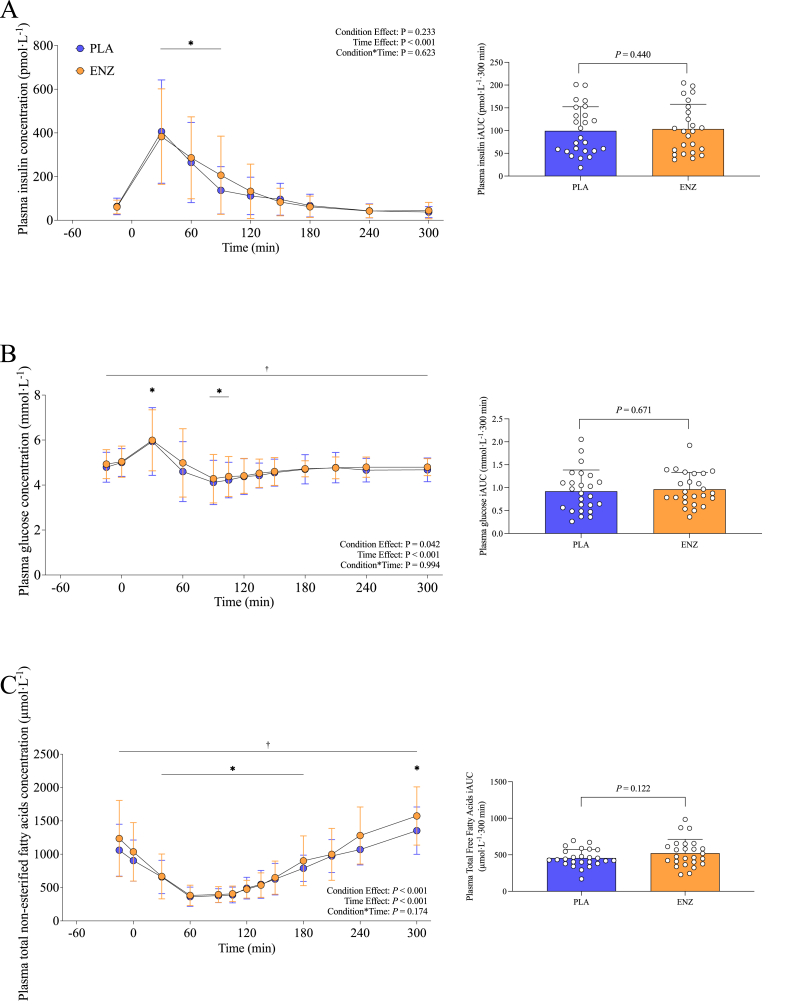
Plasma insulin (A), glucose (B), and total free fatty acids (C) mean postprandial time series concentrations (insulin in picomoles per liter per 300 minutes; glucose in millimoles per·liter per 300 minutes; and total free fatty acids in micromoles per liter·per 300 minutes) of participants who consumed a mixed meal with PLA or ENZ in a crossover clinical trial. Corresponding insets show iAUC concentrations (insulin in picomoles per liter per 300 minutes; glucose in millimoles per·liter per 300 minutes; and total free fatty acids in micromoles per liter·per 300 minutes). ∗Denotes significant effect of time (*t* = –15; *P* < 0.05). ^†^Denotes significant effect of treatment (*P* < 0.05). ENZ, mixture of 6 microbial enzyme preparations; iAUC, incremental AUC; PLA, placebo.

Plasma insulin concentrations increased, regardless of treatment, during the postprandial period (*P* < 0.001). However, no statistical significance was observed for treatment, treatment × time, mean iAUC (in picomoles per liter per 300 minutes), *T*_max_ (in minutes), or *C*_max_ (in picomoles per liter per 300 minutes) (all *P* > 0.05) (Figure 4A; Supplemental Table 2).

#### Glucose

Plasma glucose concentrations increased after mixed meal ingestion with ENZ and PLA (*P* < 0.001) and were amplified to a greater degree with ENZ than with PLA (*P* = 0.042) (Figure 4B). In contrast, no statistical significance was observed for treatment × time, mean iAUC (in millimoles per liter per 300 minutes), *T*_max_ (in minutes), or *C*_max_ (millimoles per liter per·300 minutes) (all *P* > 0.05) (Figure 4B; Supplemental Table 2).

#### Total NEFA

Plasma total NEFA concentrations increased, regardless of treatment, during the postprandial period (*P* < 0.001) and were elevated to a significantly greater extent with ENZ than with PLA (*P <* 0.001), despite no statistical significance being observed for treatment × time (*P* = 0.174) (Figure 4C). Additionally, total NEFA *C*_max_ was significantly greater (*P =* 0.006) with ENZ (1694.9 ± 471.7 μmol/L/300 min) compared with PLA (1431.3 ± 334.6 μmol/L/300 min) (Supplemental Table 2). Conversely, statistically significance was not observed for total NEFA *T*_max_ (in minutes) between ENZ and PLA (*P* > 0.05) (Supplemental Table 2).

### Secondary endpoints (additional)

#### Appetite

**FIGURE 5:**
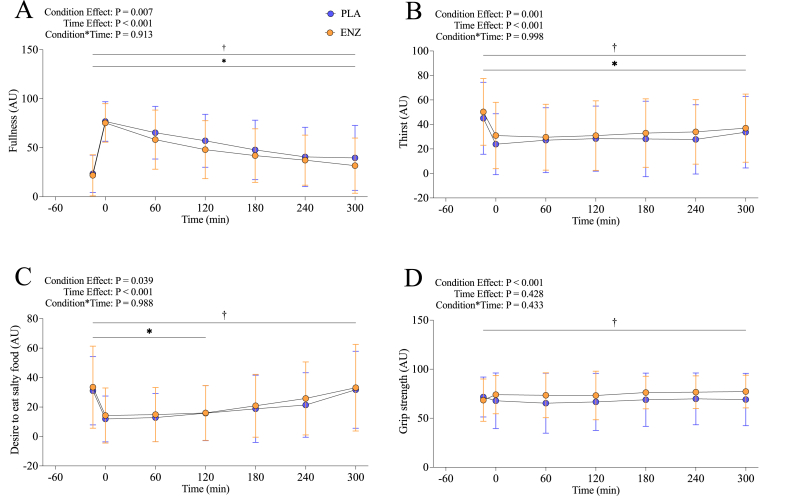
Self-reported feelings of fullness (A), thirst (B), desire to eat salty food (C), and grip strength (D) collected via a visual analog scale (VAS) questionnaire during the postabsorptive and postprandial periods (1·300 min) for participants who consumed a mixed meal with PLA or ENZ in a crossover clinical trial. ∗Denotes significant effect of time (*t* = –15; *P* < 0.05). ^†^Denotes significant effect of treatment (*P* < 0.05). AU, arbitrary units; ENZ, mixture of 6 microbial enzyme preparations; PLA, placebo.

Of the 12 VAS scores assessed, 9 were significantly affected by time (specific measures highlighted in Table 3; *P* < 0.05) and 4 were affected by treatment. Subjective fullness was significantly lower with ENZ compared with PLA (*P* = 0.007). Subjective thirst, desire for salty food, and grip strength were each significantly higher with ENZ compared with PLA (*P* = 0.002, *P* = 0.039, and *P* = 0.001, respectively). No statistical significance was observed for treatment × time for any measure (all *P* > 0.05) (Figure 5A–D; Table 3).

**TABLE 3:** Subjective visual analog scale scores taken during postabsorptive (1 measure taken) and postprandial (6 measures taken hourly; 300 min) periods of participants who consumed a mixed meal with a mixture of 6 microbial enzyme preparations (ENZ) or placebo (PLA) in a crossover clinical trial

Measure (AU)	PLA	ENZ	All	*P* value
Fullness^1^	49.9 ± 12.9	43.5 ± 10.8	46.7 ± 14.8	0.007
Hunger^1^	28.4 ± 14.2	31.1 ± 13.5	29.7 ± 13.5	0.294
Desire to eat^1^	32.8 ± 24.5	33.6 ± 26.2	33.2 ± 25.4	0.913
Prospective food consumption (AU)∗	35.7 ± 11.6	36.6 ± 10.2	36.2 ± 13.4	0.803
Preoccupation with food^1^	19.5 ± 11.9	22.3 ± 13.6	20.9 ± 12.2	0.451
Thirst^1^	29.9 ± 16.2	35.8 ± 15.2	32.8 ± 15.3	0.002
Desire for salty food^1^	21.4 ± 13.3	24.7 ± 15.3	23.0 ± 12.4	0.039
Desire for fatty food^1^	15.2 ± 11.2	15.5 ± 10.9	15.3 ± 10.4	0.688
Desire for sweet food^1^	29.2 ± 15.9	28.1 ± 15.0	28.7 ± 15.1	0.307
Shakiness of hands	5.2 ± 7.1	6.6 ± 11.4	5.9 ± 6.9	0.487
Grip strength	67.2 ± 12.0	72.4 ± 11.8	69.8 ± 12.9	0.001
Itchiness of scalp	5.6 ± 12.1	4.7 ± 9.5	5.2 ± 10.8	0.707

Data are mean ± SD. *P* value represents main effect of treatment.

Abbreviation: AU, arbitrary units.

1 Denotes significant effect of time (*P* < 0.05).

#### Gastrointestinal symptoms and bowel habits

Across the 3-wk supplementation periods, various gastrointestinal symptoms were assessed daily using the BF-GITFQ questionnaire, and no statistically significant difference was observed for time, treatment, or treatment × time (all *P* > 0.05; data not shown). Of the various weekly gastrointestinal symptoms assessed by the GITQ, no statistically significant difference was observed for treatment (all *P* > 0.05; data not shown). Of the various bowel movement measures assessed daily by the BF-GITFQ questionnaire, no statistical significance was observed for time, treatment, or treatment × time (all *P* > 0.05; data not shown).

#### Sleep

Across the 3-wk supplementation periods, sleep quality was assessed by a weekly single-item SI-SQS questionnaire, which demonstrated no statistical significance for time, treatment, or treatment × time (*P* > 0.05; data not shown).

### Exploratory (post hoc)

#### Individual postprandial amino acid responses and enzyme response modeling

**FIGURE 6:**
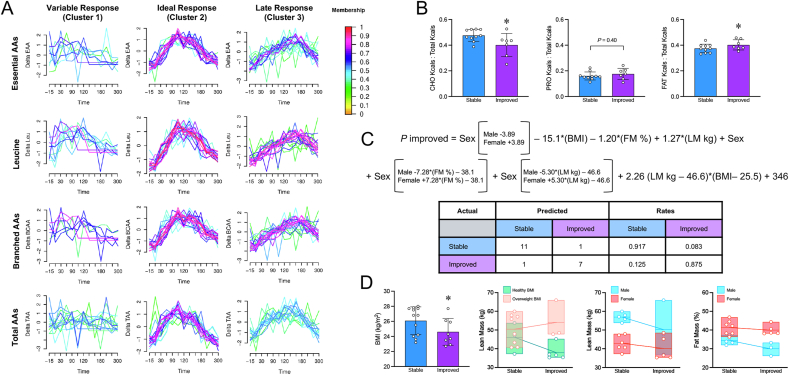
Soft clustering via MFuzz reveals 3 distinct postprandial amino acid responses across all amino acid categories (A). Macronutrient intake between those who experienced improved postprandial amino acids responses with ENZ compared with those who were stable (B). Logistic regression equation and ability to predict stable compared with improved responses to ENZ (C). Plots of interaction terms in the logistic regression model (D). ∗*P* < 0.05. AA, amino acid; BCAA, branched chain amino acids; CHO, carbohydrates; EAA, essential amino acids; FM, fat mass; LM, lean mass; TAA, total amino acids.

Soft clustering of postprandial amino acids consistently yielded 3 clusters: *1*) cluster 1 – “variable” response; *2*) cluster 2 – “ideal” response; and *3*) cluster 3 – “late” response (Figure 6A). Group membership proportions resulting from the soft clustering analyses are reported in the Supplemental Methods (Supplemental Table 3). Among all samples, 20% (10/50) exhibited a variable response, 44% (22 of 50) exhibited an ideal response, and 36% (18/50) exhibited a late response. Although participant postprandial circulating amino acid responses in cluster 3 had later *T*_*max*_ values, they did not significantly differ in *T*_*max*_ concentrations compared with those in cluster 2 (EAA cluster, *P* = 0.32; leucine cluster, *P* = 0.74, BCAA cluster, *P* = 0.50; TAA cluster, *P* = 0.26). Comparing within each individual across treatments, 32% (8/25) improved their postprandial amino acid response with ENZ, 28% (7/25) had an ideal amino acid response that remained stable across treatments, 20% (5/25) had a late amino acid response that remained stable across treatments, and 20% (5/25) had ideal or late amino acid responses that worsened with ENZ (these participants were excluded from further analyses). Compared with individuals with stable responses, those with improved responses demonstrated no statistical differences in age (*P* = 0.89), percentage fat mass (*P* = 0.58), kilograms lean mass (*P* = 0.18), habitual total kilocalorie intake (*P* = 0.89), habitual protein energy intake (*P* = 0.40), or habitual fiber intake per total kilocalories (*P* = 0.36). However, individuals with improved responses had a lower proportion of total kilocalories from carbohydrate (*P* = 0.020), higher proportion of total kilocalories from fat (*P* = 0.049), and lower BMI (*P* = 0.046) compared with individuals with stable responses (Figure 6B). Although potentially important, nutrition data were not included in further analyses due to missing data.

Logistic regression was conducted to understand what individual factors contribute to ENZ responsivity. The equations for the final model and its prediction accuracy are displayed in Figure 6C, with the following terms: sex × body fat (as percentage; *P* < 0.00001), sex × lean mass (in kilograms; *P* = 0.00004), BMI × lean mass (in kilograms; *P* = 0.00007), BMI (*P* = 0.012), body fat (as percentage; *P* = 0.94), lean mass (in kilograms; *P* = 0.95), and sex (*P* = 0.95). Whole model test and lack of fit χ^2^ were *P* = 0.0028 and *P* = 0.95, respectively. The overall accuracy of the model was 90%, only inaccurately predicting ENZ responsivity in 1 individual per class. Although individual statistics were not conducted on interaction terms, their numerical relationships are displayed in Figure 6D. Overall, individuals with overweight BMI categorization with higher lean mass, females (but not males) with lower lean mass, and males (but not females) with lower fat mass percentage seemed more likely to respond to ENZ supplementation.

### Adverse events

Six of the 30 enrolled participants reported a total of 6 adverse events. Three adverse events (i.e., 2 instances of COVID-19 diagnosis and 1 instance of allergy-related headache) were deemed acute, of mild severity, and not related to the treatment product. The remaining 3 adverse events were reported during the ENZ supplementation period and deemed potentially related to the treatment product, including *1*) a moderately severe report of intermittent abdominal discomfort (4 d duration); *2*) a mildly severe report of acute general discomfort (6 d duration); and *3*) a moderately severe report of intermittent diarrhea and sleep disturbance, as well as persistent abdominal discomfort (21 d duration). All participants were given the option to continue onward or discontinue participation. All participants decided to continue with enrollment. All adverse events were deemed not serious, and all participants recovered from any self-reported symptoms. Despite 3 gastrointestinal-related adverse events potentially related to ENZ, and none related to PLA, no significant effects of treatment on subjective gastrointestinal symptoms were observed for daily or weekly measures (as previously noted) (all *P* > 0.05; data not shown).

## Discussion

Past in vitro work demonstrated that a specific supplemental enzyme mixture can effectively compensate for age-related decline in digestive function [21]. This study sought to extend this in vitro study into a clinical trial to investigate the effectiveness of enzyme supplementation as a potential strategy for augmenting postprandial nutrient bioavailability in middle-aged and older adults. The efficacy of enzyme supplementation was investigated by examining the postprandial effects of consuming a mixed meal with a supplemental mixture of 6 enzyme preparations (ENZ). Although there were no statistical differences in plasma amino acid concentrations or net amino acid exposure (iAUC) over the 5-h postprandial period, acute ENZ supplementation demonstrated significantly faster time to maximum concentration (*T*_*max*_) of leucine and a trend toward faster EAA *T*_*max*_ (*P* = 0.089) compared with placebo (PLA) (Table 2). In addition, ENZ administration was associated with increased postprandial plasma glucose and total NEFA concentrations compared with PLA. Subjective appetite-related measures also demonstrated feelings of reduced fullness, increased thirst, increased desire for salty foods, and increased perceived grip strength with ENZ compared with PLA.

Our mixed meal approach in middle-aged and older adults is novel, as prior clinical trials to test enzyme supplementation on modulating postprandial plasma nutrient concentrations have enrolled younger adults and tested proteases with isolated protein sources as the test substrate [[38], [39], [40], [41], [42]]. Although we did not observe a significantly greater increase in postprandial amino acid concentrations with enzyme supplementation during the 5-h postprandial period (EAAs, *P* = 0.099; TAAs*, P* = 0.082) or total net amino acid exposure (i.e., iAUC), the significant findings identified for leucine (see *T*_max_ values for ENZ compared with PLA; Table 3) suggest enzyme supplementation may have a positive impact on modulating protein digestion and amino acid absorption kinetics for middle-aged and older adults. Specifically, we showed postprandial time to peak plasma leucine concentrations were faster in ENZ (*T*_max_ = 121 ± 56 min) compared with PLA (141 ± 49 min) with no statistical differences in *C*_max_ between the 2 trials. Past research efforts have clearly demonstrated that a “fast” leucine response, or trigger, serves as an independent regulatory factor for the stimulation of postprandial muscle protein synthesis rates [57,59]. Accordingly, clinical feeding formulas often incorporate leucine to leverage its role as a potent anabolic signaling molecule, in addition to being a substrate, to stimulate muscle protein synthesis and remodeling to support lean mass preservation. Our findings point toward an innovative strategy to enhance the age-related anabolic action of a mixed meal, particularly because the postprandial release of dietary amino acids with mixed meal ingestion is more prolonged, minimizing the peak amplitude of the leucine anabolic signal when compared with isolated protein sources. However, this notion would require more rigorous testing through direct measurements of whole-body substrate kinetics and muscle protein synthesis rates via stable isotopes and/or longer-term clinical trials to determine functional health outcomes.

As the ENZ mixture was designed to promote digestion of all macronutrients, postprandial plasma total NEFA and glucose were also measured. ENZ supplementation elevated both postprandial plasma total NEFA and glucose concentrations. The efficacy of lipase, particularly porcine pancreatic lipase, has traditionally been evaluated by its ability to improve the coefficient of fat absorption, an outcome determined by the relationship between dietary fat intake and fecal fat excretion [60]. To our knowledge, this study of ENZ is the first to show improved postprandial plasma total NEFA concentrations after lipase administration, serving as an alternate marker of increased fat digestion. Future research should extend postprandial measurements to 6–8 h to more accurately capture the time-dependent relationship between plasma total NEFA and also measure plasma triglycerides [61]. The significant increase in plasma glucose concentrations in the ENZ group is corroborated by similar clinical findings demonstrating that a distinct multienzyme mixture is capable of increasing carbohydrate digestion [60]. These outcomes are notable considering dietary guidelines generally advocate for carbohydrate intake to account for at least half of daily ingested calories, yet insufficient digestion and absorption of carbohydrates may lead to gastrointestinal distress, including gas, bloating, nausea, abdominal pain, and diarrhea [62,63]. A possible contributor to these gastrointestinal symptoms is the rate of gastric emptying. Although gastric emptying time was not measured in this study, subjective appetite measures can serve as a proxy, as discussed in the next paragraph.

We observed significant findings within several gastrointestinal-related measures for 4 of the 12 appetite VAS questions: *1*) “How strong is your feeling of fullness?”, *2*) “How strong is your feeling of thirst?”, *3*) “How strong is your desire to eat something salty?”, and *4*) “How strong is your grip?”. These statistically significant differences emerged despite a wide range of subjective responses (i.e., as evidenced by our wide error bars). The first, fullness, is particularly relevant to gastric emptying, which depends on several physiochemical properties of the meal, such as composition, viscosity, volume, caloric density, etc. Gastrointestinal symptoms such as gas and nausea may lead to a feeling of fullness and impair appetite, which would be particularly problematic for older adults; indeed, such alterations likely contribute to reduced food intake thereby depriving the body of nutrients that are paramount to maintain health and physical performance during aging [64,65]. Our results of reduced feelings of perceived fullness after ENZ supplementation are coupled with a reported increase in thirst and an increased desire to eat something salty. Though these 2 outcomes appear capable of working together to improve hydration and electrolyte balance, thereby possibly helping stimulate appetite to increase overall nutritional intake, recent work does not currently support this notion. Temperance of these 2 VAS outcomes is prudent, yet our observation of reduced feelings of fullness still might suggest that the ENZ treatment can promote augmented nutritional intake to help older adults overcome common age-related challenges, such as reduced food intake and reduced speed of gastric emptying [66]. This, in turn, could facilitate positive downstream outcomes to support healthy aging. Further assessment of this relationship between supplemental enzymes and appetite regulation would benefit from utilizing ad libitum dietary feeding patterns in their methodological design. Personalized and precision nutrition are growing fields aiming to provide tailored dietary recommendations to individuals and specific subpopulations, respectively [67]. Unlike traditional mean-focused analyses, our data-driven approach allowed us to identify distinct postprandial amino acid responses. Particularly, this approach allowed us to identify specific characteristics of individuals who benefited most from ENZ supplementation. Importantly, our results point to certain subgroups of middle-aged and older adults who may benefit more from ENZ supplementation in terms of modulating changes in postprandial plasma amino acid concentrations. Specifically, our results suggest that adults with higher BMI (particularly for males and those with lower lean mass) may be more likely to benefit from ENZ supplementation [68]. These individuals with a higher BMI may not readily benefit from the previously noted supposition that supplemental enzymes could augment total dietary intake habits (i.e., individuals with higher BMI would have less to gain in this regard); they may, however, still be able to further gain from a more potent muscle protein synthetic response to resistance training that results from enhanced EAA absorption kinetics. Our understanding of how adiposity affects protein digestion and amino acid absorption kinetics with mixed meal feedings in adults with overweight or obesity remains limited, largely due to the specialized tools required for more direct assessment of the postprandial release of dietary amino acids into circulation [69]. Nonetheless, further studies are required to understand the potential of ENZ to improve postprandial amino acid profiles in people with overweight or obesity and ultimately help resolve these data. Although there is a well documented decrease in digestive efficiency with age, it is perhaps unsurprising that age was not a strong determinant of ENZ benefit given the narrow age range of recruited participants (e.g., 56 ± 11 y) included in the current study [56]. Clearly, a future study with a similar design across a wider age continuum would help clarify how age is a determinant of ENZ improvement. Establishing this relationship would provide more nuance to recent findings that demonstrated that age-related differences in plasma amino acid concentrations are likely influenced by other factors such as plasma amino acid distribution volume (major factor), habitual diet, muscle quantity/quality, insulin resistance, and inflammation [70]. Although our regression model could predict ENZ responsivity highly accurately from a small amount of easily obtainable data, it would also further benefit from inclusion of other data, particularly longitudinal nutrient intake and digestive symptoms that may influence small intestinal adaption and nutrient processing [71,72]. Therefore, the cluster-associated regression analyses should be considered hypothesis-generating, but supported by other emergent literature, and requires validation and refinement using a larger cohort [73,74].

This study has limitations. Our power analysis served as a design-stage approximation rather than an exact model parameterization. Specifically, our calculation for the primary endpoints was created based on prior work by Jäger et al. [48] but differed in that we tested enzymes (not probiotics), utilized a mixed meal (instead of an isolated protein source), and assessed iAUC (rather than AUC). Further, we acknowledge our analysis utilized mixed effects modeling despite assuming a 2-tailed *t*-test in power calculation, likely underestimating the required sample size. The determined sample of *n* = 27 participants deemed necessary to detect significant differences between treatments was also slightly underpowered as 5 of the 30 enrolled participants were removed from the final analysis set due to critical protocol deviations. Next, our participants were aged 56 ± 11 y, and the inclusion of adults exclusively over 60 or 65 y may provide greater insight on the utility of supplemental enzymes to mitigate digestive senescence. In terms of the mixed meal tested, the high amount of protein (34 g) and high-quality protein sources (chicken, milk) included may have minimized the potential effects of enzymes to further potentiate postprandial plasma amino acid concentrations. The “static” determination of plasma concentrations following this meal also provides no insight into the flux of substrate(s) (i.e., rates of appearance, disappearance, etc.), which precludes the ability to comment on the metabolic fates of the nutrients that were consumed. Finally, acute human metabolic studies such as the present study provide a framework for longer duration interventions, which are warranted to better understand the significance of enzyme supplementation on health and physical performance outcomes for adults who are advancing in age.

In conclusion, this clinical trial provides novel insights into the capabilities of enzyme supplementation to modulate postprandial plasma nutrient concentrations after consumption of a mixed meal in healthy middle-aged and older adults. Supplementation of a mixed meal with 6 enzymes enhanced the time to peak plasma leucine concentrations and elevated postprandial plasma glucose and total NEFA concentrations in adults with advancing age. The translation of these acute metabolic effects to longer-term health outcomes requires further testing. Nonetheless, dietary supplementation with enzymes appears to be a safe and well-tolerated candidate strategy to improve nutrient bioavailability in healthy middle-aged and older adults.

## Author contributions

The authors’ responsibilities were as follows – NAB, SMG: contributed to the conception and design of the experiments; MTD, NAB, BRL: contributed to drafting or revising the intellectual content of the manuscript and had primary responsibility for the final content; MTD, ATA, TMB, ŽZ, JWW: contributed to the collection of data; MTD, NAB, ATA, BRL, DAA, ŽZ, SMG, AVU, HDH: contributed to the analysis and interpretation of data; and all authors: read and approved the final manuscript.

## Data availability

Data described in the manuscript, code book, and analytic code will be made available upon request pending approval from the principal investigator.

## Funding

This study was funded by BIO-CAT, Inc.

## Conflict of interest

This project was funded by BIO-CAT, Inc., which provided the investigational products, including the mixture of 6 enzyme preparations (Trade Name: OPTIZIOME Macro Digest). BIO-CAT, Inc. has previously filed a patent application related to compositions and uses described in this manuscript (United States Patent Application No. 18/688,983). SMG was an employee of BIO-CAT, Inc. at the time of study and was involved in the design of the study, in the interpretation of data, in the editing of the manuscript, and in the decision to publish the results. SMG is the current founder at SAPIOME LLC, which seeks to support biotech, food, and supplement businesses. HDH is a member of *The Journal of Nutrition* Editorial Board. The authors declare no other conflicts of interest.
