# Supplementary material for: Oral Multienzyme Supplementation Alters Postprandial Plasma Nutrient Concentrations after a Mixed Meal in Healthy Middle-Aged and Older Adults: A Randomized, Double-Blind, Placebo-Controlled, Crossover Trial
Source: J Nutr. 2026 Feb 7;156(4):101400. doi: 10.1016/j.tjnut.2026.101400 (PMC13084569; doi:10.1016/j.tjnut.2026.101400)
Supplement: Multimedia component 1 [file mmc1.docx]

**SUPPLEMENTAL TABLES**

| **Supplemental Table 1.** Composition of the mixture of six microbial enzyme preparations (ENZ) consumed by participants in a randomized, placebo-controlled, crossover clinical trial.^1^ | | | | | |
| --- | --- | --- | --- | --- | --- |
| **Primary Enzyme**  **Activity** | **CAS No.** | **EC No.** | **Source Organism** | **Activity per dose^2^** | **Optimum pH**  **(Range)^3^** |
| Protease | 9025-49-4 | 3.4.23.18 | *Aspergillus oryzae* | 60,000 HUT | 3.0 (3.0–6.0) |
| Protease | 9025-49-4 | 3.4.23.18 | *Aspergillus niger* | 300 SAP | 3.0 (1.5–4.5) |
| Protease | 9074-07-1 | 3.4.21.63 | *Aspergillus melleus* | 50 LAPU | 7.5 (5.5–10.0) |
| Lipase | 9001-62-1 | 3.2.1.3 | *Candida cylindracea* | 3000 FIP | 7.0 (3.0–9.0) |
| Amylase | 9000-90-2 | 3.2.1.1 | *Aspergillus oryzae* | 10,000 SKB^4^ | 5.0 (3.5–6.5) |
| Glucoamylase | 9032-08-0 | 3.2.1.3 | *Aspergillus niger* | 25 AGU | 4.5 (3.0–6.0) |
| AGU, amyloglucosidase unit; CAS No., Chemical Abstract Society Registry No.; EC No., Enzyme Commission No.; FIP, Fédération Internationale Pharmaceutique unit; HUT, hemoglobin unit tyrosine base; LAPU, leucine aminopeptidase unit; SAP, spectrophotometric acid protease unit; SKB, Sandstedt, Kneen, and Blish method. | | | | | |

**Footnotes:** ^1^ Inactive ingredients include potato dextrin, tapioca maltodextrin, dextrose, calcium carbonate, potassium sorbate, sodium benzoate, and silicon dioxide. ^2^ Enzyme activity assays used to separately confirm the activity of each enzyme preparation were previously described.^28^ The listed activity is specific to the individual enzyme preparation added to the mixture. The total activities of the ENZ mixture may be higher than listed due to the “side activities” of the individual enzyme preparations. For example, ENZ routinely shows proteolytic activity ≥ 80,000 HUT. ^3^ The values are according to the manufacturer or supplier’s technical documentation based on various assays that are amenable to pH adjustment. Bounds of the optimal pH range are defined by ≥50% optimal activity. ^4^ One SKB unit is equivalent to one dextrinizing unit (DU). Abbreviations: AGU—amyloglucosidase unit; CAS No.—Chemical Abstract Society Registry No.; EC No.—Enzyme Commission No.; FIP—Fédération Internationale Pharmaceutique unit; HUT—hemoglobin unit tyrosine base; LAPU—leucine aminopeptidase unit; SAP—spectrophotometric acid protease unit; SKB—Sandstedt, Kneen, and Blish method.

| **Supplemental Table 2.** Postprandial plasma concentration incremental area under the curve (iAUC), maximum concentration (C_max_), and time to maximum concentration (T_max_) for total free fatty acids (FFA), glucose, and insulin of participants who consumed a mixed meal with a mixture of six microbial enzyme preparations (ENZ) or placebo (PLA) in a crossover clinical trial. | | | | | |
| --- | --- | --- | --- | --- | --- |
|  |  | **ENZ** | **PLA** | ***P*-value** | **Cohen's *d*** |
| FFA | iAUC | N/A | N/A | N/A | N/A |
|  | *C*_max_^2^ | 1694.9 ± 471.7 | 1431.3 ± 334.6 | 0.006 | 0.126 |
|  | *T*_max_^3^ | 234 ± 115.7 | 223.2 ± 124.8 | 0.753 | -0.361 |
| Glucose | iAUC^1^ | 0.98 ± 0.36 | 0.92 ± 0.46 | 0.671 | -0.067 |
|  | *C*_max_^2^ | 6.24 ± 1.15 | 6.29 ± 1.36 | 0.799 | 0.181 |
|  | *T*_max_^3^ | 36 ± 34.1 | 26.4 ± 31.3 | 0.096 | -0.157 |
| Insulin | iAUC^4^ | 103.5 ± 54.4 | 98.5 ± 53.9 | 0.44 | -0.039 |
|  | *C*_max_^5^ | 444.2 ± 221.2 | 431.4 ± 229.4 | 0.658 | 0.128 |
|  | *T*_max_^3^ | 43.8 ± 21.6 | 37.5 ± 20.3 | 0.135 | 0.027 |
| Data are mean ± SD. FFA, total free fatty acids; ^1^ Units for iAUC (glucose) are mmol·L^-1^·300 min^-1^; ^2^ Units for *C*_max_ (FFA and glucose) are mmol·L^-1^; ^3^ Units for *T*_max_ are min; ^4^ Units for iAUC (insulin) are pmol·L^-1^·300 min^-1^; ^5^ Units for *C*_max_ (insulin) are pmol·L^-1^. | | | | | |

| **Supplemental Table 3.** Postprandial amino acid response group membership proportions of soft clustering via MFuzz of participants who consumed a mixed meal with a mixture of six microbial enzyme preparations (ENZ) or placebo (PLA) in a crossover clinical trial. Three distinct postprandial amino acid response clusters emerged across all amino acid categories: 1 (‘**^1^**’) = variable response; 2 (‘**^2^**’) = ideal response; 3 (‘**^3^**’) = late response. | | | | | | | | | | | | | |
| --- | --- | --- | --- | --- | --- | --- | --- | --- | --- | --- | --- | --- | --- |
| **Participant** | **Treatment** | **Leu^1^** | **Leu^2^** | **Leu^3^** | **BCAA^1^** | **BCAA^2^** | **BCAA^3^** | **EAA^1^** | **EAA^2^** | **EAA^3^** | **TAA^1^** | **TAA^2^** | **TAA^3^** |
| 008 | ENZ | 0.265 | 0.367 | 0.368 | 0.315 | 0.300 | 0.384 | 0.320 | 0.311 | 0.369 | 0.244 | 0.328 | 0.428 |
| 016 | ENZ | 0.769 | 0.037 | 0.194 | 0.036 | 0.724 | 0.241 | 0.048 | 0.718 | 0.234 | 0.030 | 0.698 | 0.272 |
| 020 | ENZ | 0.899 | 0.032 | 0.069 | 0.028 | 0.870 | 0.101 | 0.056 | 0.739 | 0.205 | 0.090 | 0.534 | 0.376 |
| 028 | ENZ | 0.132 | 0.769 | 0.099 | 0.821 | 0.098 | 0.080 | 0.731 | 0.145 | 0.124 | 0.591 | 0.216 | 0.192 |
| 031 | ENZ | 0.924 | 0.030 | 0.046 | 0.026 | 0.908 | 0.066 | 0.028 | 0.890 | 0.082 | 0.025 | 0.783 | 0.192 |
| 034 | ENZ | 0.741 | 0.033 | 0.226 | 0.018 | 0.825 | 0.157 | 0.031 | 0.768 | 0.201 | 0.046 | 0.676 | 0.278 |
| 035 | ENZ | 0.094 | 0.012 | 0.895 | 0.029 | 0.247 | 0.725 | 0.068 | 0.297 | 0.635 | 0.251 | 0.377 | 0.372 |
| 050 | ENZ | 0.827 | 0.062 | 0.112 | 0.030 | 0.828 | 0.143 | 0.034 | 0.860 | 0.106 | 0.023 | 0.823 | 0.154 |
| 052 | ENZ | 0.534 | 0.069 | 0.397 | 0.055 | 0.441 | 0.504 | 0.125 | 0.394 | 0.481 | 0.145 | 0.354 | 0.501 |
| 055 | ENZ | 0.604 | 0.049 | 0.347 | 0.028 | 0.364 | 0.608 | 0.066 | 0.516 | 0.418 | 0.056 | 0.540 | 0.404 |
| 060 | ENZ | 0.243 | 0.021 | 0.736 | 0.013 | 0.191 | 0.796 | 0.032 | 0.251 | 0.717 | 0.060 | 0.323 | 0.616 |
| 062 | ENZ | 0.679 | 0.238 | 0.083 | 0.241 | 0.644 | 0.115 | 0.153 | 0.720 | 0.127 | 0.031 | 0.829 | 0.140 |
| 067 | ENZ | 0.886 | 0.036 | 0.079 | 0.048 | 0.818 | 0.133 | 0.065 | 0.740 | 0.194 | 0.095 | 0.541 | 0.364 |
| 073 | ENZ | 0.085 | 0.017 | 0.898 | 0.021 | 0.125 | 0.854 | 0.069 | 0.154 | 0.777 | 0.156 | 0.245 | 0.599 |
| 074 | ENZ | 0.197 | 0.707 | 0.096 | 0.692 | 0.212 | 0.096 | 0.469 | 0.350 | 0.182 | 0.353 | 0.359 | 0.288 |
| 077 | ENZ | 0.954 | 0.023 | 0.024 | 0.018 | 0.936 | 0.046 | 0.025 | 0.912 | 0.063 | 0.023 | 0.826 | 0.151 |
| 079 | ENZ | 0.707 | 0.018 | 0.275 | 0.011 | 0.617 | 0.372 | 0.012 | 0.799 | 0.189 | 0.017 | 0.555 | 0.428 |
| 082 | ENZ | 0.285 | 0.590 | 0.126 | 0.657 | 0.194 | 0.149 | 0.644 | 0.190 | 0.167 | 0.597 | 0.192 | 0.211 |
| 085 | ENZ | 0.162 | 0.029 | 0.810 | 0.029 | 0.143 | 0.829 | 0.132 | 0.245 | 0.623 | 0.232 | 0.332 | 0.437 |
| 086 | ENZ | 0.256 | 0.022 | 0.721 | 0.028 | 0.363 | 0.609 | 0.048 | 0.497 | 0.456 | 0.071 | 0.416 | 0.513 |
| 087 | ENZ | 0.357 | 0.260 | 0.383 | 0.264 | 0.339 | 0.397 | 0.298 | 0.302 | 0.400 | 0.357 | 0.288 | 0.354 |
| 090 | ENZ | 0.545 | 0.321 | 0.134 | 0.117 | 0.715 | 0.168 | 0.094 | 0.670 | 0.236 | 0.097 | 0.469 | 0.433 |
| 100 | ENZ | 0.399 | 0.477 | 0.124 | 0.227 | 0.520 | 0.253 | 0.339 | 0.410 | 0.251 | 0.244 | 0.370 | 0.386 |
| 107 | ENZ | 0.234 | 0.562 | 0.203 | 0.679 | 0.171 | 0.150 | 0.612 | 0.187 | 0.201 | 0.574 | 0.199 | 0.226 |
| 109 | ENZ | 0.194 | 0.067 | 0.740 | 0.123 | 0.247 | 0.630 | 0.257 | 0.240 | 0.503 | 0.385 | 0.219 | 0.396 |
| 008 | PLA | 0.178 | 0.156 | 0.666 | 0.158 | 0.195 | 0.647 | 0.336 | 0.195 | 0.469 | 0.527 | 0.170 | 0.304 |
| 016 | PLA | 0.812 | 0.114 | 0.074 | 0.122 | 0.746 | 0.132 | 0.168 | 0.661 | 0.171 | 0.133 | 0.584 | 0.283 |
| 020 | PLA | 0.466 | 0.153 | 0.381 | 0.124 | 0.488 | 0.389 | 0.175 | 0.347 | 0.477 | 0.212 | 0.333 | 0.455 |
| 028 | PLA | 0.089 | 0.030 | 0.881 | 0.049 | 0.170 | 0.781 | 0.135 | 0.241 | 0.624 | 0.222 | 0.329 | 0.449 |
| 031 | PLA | 0.198 | 0.145 | 0.656 | 0.200 | 0.217 | 0.583 | 0.398 | 0.183 | 0.419 | 0.575 | 0.162 | 0.263 |
| 034 | PLA | 0.497 | 0.053 | 0.450 | 0.052 | 0.495 | 0.453 | 0.106 | 0.417 | 0.478 | 0.135 | 0.348 | 0.517 |
| 035 | PLA | 0.180 | 0.094 | 0.725 | 0.171 | 0.242 | 0.587 | 0.314 | 0.245 | 0.441 | 0.486 | 0.210 | 0.304 |
| 050 | PLA | 0.121 | 0.019 | 0.860 | 0.016 | 0.148 | 0.836 | 0.051 | 0.216 | 0.733 | 0.076 | 0.350 | 0.574 |
| 052 | PLA | 0.323 | 0.498 | 0.179 | 0.298 | 0.469 | 0.233 | 0.376 | 0.332 | 0.292 | 0.429 | 0.270 | 0.301 |
| 055 | PLA | 0.971 | 0.011 | 0.018 | 0.008 | 0.956 | 0.035 | 0.020 | 0.925 | 0.055 | 0.017 | 0.833 | 0.150 |
| 060 | PLA | 0.063 | 0.008 | 0.929 | 0.011 | 0.100 | 0.888 | 0.031 | 0.172 | 0.797 | 0.075 | 0.365 | 0.561 |
| 062 | PLA | 0.235 | 0.661 | 0.104 | 0.668 | 0.206 | 0.126 | 0.668 | 0.206 | 0.126 | 0.424 | 0.289 | 0.286 |
| 067 | PLA | 0.931 | 0.036 | 0.033 | 0.020 | 0.916 | 0.064 | 0.029 | 0.896 | 0.075 | 0.025 | 0.790 | 0.185 |
| 073 | PLA | 0.845 | 0.016 | 0.140 | 0.019 | 0.676 | 0.306 | 0.031 | 0.629 | 0.340 | 0.037 | 0.512 | 0.451 |
| 074 | PLA | 0.346 | 0.582 | 0.071 | 0.267 | 0.589 | 0.144 | 0.346 | 0.505 | 0.149 | 0.127 | 0.624 | 0.250 |
| 077 | PLA | 0.335 | 0.057 | 0.607 | 0.029 | 0.319 | 0.652 | 0.070 | 0.535 | 0.395 | 0.052 | 0.718 | 0.230 |
| 079 | PLA | 0.069 | 0.028 | 0.903 | 0.028 | 0.090 | 0.883 | 0.046 | 0.113 | 0.841 | 0.070 | 0.319 | 0.611 |
| 082 | PLA | 0.734 | 0.225 | 0.041 | 0.093 | 0.840 | 0.067 | 0.097 | 0.813 | 0.090 | 0.036 | 0.812 | 0.152 |
| 085 | PLA | 0.297 | 0.082 | 0.621 | 0.107 | 0.370 | 0.523 | 0.144 | 0.343 | 0.513 | 0.152 | 0.331 | 0.516 |
| 086 | PLA | 0.320 | 0.316 | 0.365 | 0.359 | 0.265 | 0.375 | 0.512 | 0.204 | 0.284 | 0.621 | 0.159 | 0.220 |
| 087 | PLA | 0.124 | 0.020 | 0.856 | 0.015 | 0.144 | 0.841 | 0.067 | 0.271 | 0.662 | 0.131 | 0.271 | 0.598 |
| 090 | PLA | 0.108 | 0.851 | 0.041 | 0.857 | 0.097 | 0.046 | 0.675 | 0.215 | 0.110 | 0.400 | 0.373 | 0.227 |
| 100 | PLA | 0.723 | 0.180 | 0.097 | 0.097 | 0.714 | 0.188 | 0.138 | 0.666 | 0.195 | 0.077 | 0.620 | 0.303 |
| 107 | PLA | 0.387 | 0.433 | 0.180 | 0.184 | 0.402 | 0.414 | 0.281 | 0.254 | 0.465 | 0.432 | 0.208 | 0.360 |
| 109 | PLA | 0.243 | 0.373 | 0.384 | 0.425 | 0.210 | 0.365 | 0.564 | 0.161 | 0.274 | 0.638 | 0.146 | 0.216 |
| 008 | ENZ | 0.265 | 0.367 | 0.368 | 0.315 | 0.300 | 0.384 | 0.320 | 0.311 | 0.369 | 0.244 | 0.328 | 0.428 |
| 016 | ENZ | 0.769 | 0.037 | 0.194 | 0.036 | 0.724 | 0.241 | 0.048 | 0.718 | 0.234 | 0.030 | 0.698 | 0.272 |
| 020 | ENZ | 0.899 | 0.032 | 0.069 | 0.028 | 0.870 | 0.101 | 0.056 | 0.739 | 0.205 | 0.090 | 0.534 | 0.376 |
| 028 | ENZ | 0.132 | 0.769 | 0.099 | 0.821 | 0.098 | 0.080 | 0.731 | 0.145 | 0.124 | 0.591 | 0.216 | 0.192 |
| 031 | ENZ | 0.924 | 0.030 | 0.046 | 0.026 | 0.908 | 0.066 | 0.028 | 0.890 | 0.082 | 0.025 | 0.783 | 0.192 |
| 034 | ENZ | 0.741 | 0.033 | 0.226 | 0.018 | 0.825 | 0.157 | 0.031 | 0.768 | 0.201 | 0.046 | 0.676 | 0.278 |
| 035 | ENZ | 0.094 | 0.012 | 0.895 | 0.029 | 0.247 | 0.725 | 0.068 | 0.297 | 0.635 | 0.251 | 0.377 | 0.372 |
| 050 | ENZ | 0.827 | 0.062 | 0.112 | 0.030 | 0.828 | 0.143 | 0.034 | 0.860 | 0.106 | 0.023 | 0.823 | 0.154 |
| 052 | ENZ | 0.534 | 0.069 | 0.397 | 0.055 | 0.441 | 0.504 | 0.125 | 0.394 | 0.481 | 0.145 | 0.354 | 0.501 |
| 055 | ENZ | 0.604 | 0.049 | 0.347 | 0.028 | 0.364 | 0.608 | 0.066 | 0.516 | 0.418 | 0.056 | 0.540 | 0.404 |
| 060 | ENZ | 0.243 | 0.021 | 0.736 | 0.013 | 0.191 | 0.796 | 0.032 | 0.251 | 0.717 | 0.060 | 0.323 | 0.616 |
| 062 | ENZ | 0.679 | 0.238 | 0.083 | 0.241 | 0.644 | 0.115 | 0.153 | 0.720 | 0.127 | 0.031 | 0.829 | 0.140 |
| 067 | ENZ | 0.886 | 0.036 | 0.079 | 0.048 | 0.818 | 0.133 | 0.065 | 0.740 | 0.194 | 0.095 | 0.541 | 0.364 |
| 073 | ENZ | 0.085 | 0.017 | 0.898 | 0.021 | 0.125 | 0.854 | 0.069 | 0.154 | 0.777 | 0.156 | 0.245 | 0.599 |
| 074 | ENZ | 0.197 | 0.707 | 0.096 | 0.692 | 0.212 | 0.096 | 0.469 | 0.350 | 0.182 | 0.353 | 0.359 | 0.288 |
| 077 | ENZ | 0.954 | 0.023 | 0.024 | 0.018 | 0.936 | 0.046 | 0.025 | 0.912 | 0.063 | 0.023 | 0.826 | 0.151 |
| 079 | ENZ | 0.707 | 0.018 | 0.275 | 0.011 | 0.617 | 0.372 | 0.012 | 0.799 | 0.189 | 0.017 | 0.555 | 0.428 |
| 082 | ENZ | 0.285 | 0.590 | 0.126 | 0.657 | 0.194 | 0.149 | 0.644 | 0.190 | 0.167 | 0.597 | 0.192 | 0.211 |
| 085 | ENZ | 0.162 | 0.029 | 0.810 | 0.029 | 0.143 | 0.829 | 0.132 | 0.245 | 0.623 | 0.232 | 0.332 | 0.437 |
| 086 | ENZ | 0.256 | 0.022 | 0.721 | 0.028 | 0.363 | 0.609 | 0.048 | 0.497 | 0.456 | 0.071 | 0.416 | 0.513 |
| 087 | ENZ | 0.357 | 0.260 | 0.383 | 0.264 | 0.339 | 0.397 | 0.298 | 0.302 | 0.400 | 0.357 | 0.288 | 0.354 |
| 090 | ENZ | 0.545 | 0.321 | 0.134 | 0.117 | 0.715 | 0.168 | 0.094 | 0.670 | 0.236 | 0.097 | 0.469 | 0.433 |
| 100 | ENZ | 0.399 | 0.477 | 0.124 | 0.227 | 0.520 | 0.253 | 0.339 | 0.410 | 0.251 | 0.244 | 0.370 | 0.386 |
| 107 | ENZ | 0.234 | 0.562 | 0.203 | 0.679 | 0.171 | 0.150 | 0.612 | 0.187 | 0.201 | 0.574 | 0.199 | 0.226 |
| 109 | ENZ | 0.194 | 0.067 | 0.740 | 0.123 | 0.247 | 0.630 | 0.257 | 0.240 | 0.503 | 0.385 | 0.219 | 0.396 |
| Three distinct postprandial amino acid responses across all amino acid categories Soft clustering via MFuzz reveals Leu, leucine; EAA, essential amino acids; BCAA, branched chain amino acids; TAA, total amino acids leucine; ^1^, cluster 1 (‘variable response’); ^2^, cluster 2 (‘ideal response’); ^3^, cluster 3 (‘late response’). | | | | | | | | | | | | | |

**SUPPLEMENTAL METHODS**

***Plasma amino acid concentrations and enrichments***

Plasma samples (25 μL) were mixed with 1) water:ACN:IPA (167 μL; 2:3:3 *v/*v), 2) (vortexed) DL-p-chlorophenylalanine (5 μL; 10ug/mL), and 3) amino acid internal standard mixture (5 μL; 10ug/mL). The aforementioned mixture was then vortexed for 10 seconds and subsequently centrifuged at 20,817 *g* and 4°C for 10 min. Supernatant (100 μL) was then removed and dried under vacuum before resuspension in 1 mL 0.1% formic acid in water prior to injection. The LC separation was performed on a Thermo Fisher Scientific Hyersil GOLD™ C18column (2.1 × 150 mm, 1.9 μm) with mobile phase A (0.1% formic acid in water) and mobile phase B (0.1% formic acid in acetonitrile) at a flow rate of 0.2 mL·min^-1^. The linear gradient was as follows: 0‒0.5 min, 0% B; 0.5‒3.5 min, 60% B; 3.5‒5.5 min, 100% B; 5.5‒7.5 min, 0% B. The autosampler and UHPLC column chamber were set at 5°C and 50°C, respectively. The injection volume was 1 μL. Mass spectra were acquired under positive electrospray ionization with the ion spray voltage of 3500 V. Selected reaction monitoring was used for the amino acid quantitation. The amino acid standard solution (Product No. AAS18; Millipore Sigma, St. Louis, MO, USA), combined with a mixture of L-tryptophan, L-glutamine, L-asparagine, and L-cysteine was used for calibration curves built in 0.5 – 5000 ng/mL range to quantify plasma amino acids. Samples were analyzed by LC-MS-MS (Altis Triple Quadrupole; Thermo Fisher Scientific Inc., Waltham, MA, USA) with TraceFinder 4.3 data acquisition software (Thermo Fisher Scientific Inc., Waltham, MA, USA).

***Plasma total free fatty acid concentrations and enrichments***

Plasma samples (20 μL) were spiked with 5μL of the internal standard mixture and extracted with methanol:chloroform (175 μL; 1:2 v/v) with BHT (2 g∙L^-1^). The mixture was then vortexed for 3 seconds and subsequently centrifuged at 20,817 *g* and 4°C for 10 min. Supernatant (100 μL) was then removed and placed within inserts prior to injection. Free fatty acids were measured by Agilent Technologies 1290 Infinity II Series (Agilent Technologies, Santa Clara, CA, USA) UPLC and 9465C Triple Quadrupole mass spectrometer in negative electrospray ionization (ESI) using MRM mode. Analytes were separated on the Acquity BEH C18 column (100 × 2.1mm, 1.7 uM; Waters, Milford, MA, USA) using a two mobile phase gradient. Mobile phase A was 5mM Ammonia acetate in water:Methanol (7:3 v/v) + 0.05% Acetic acid; mobile phase B was 5mM Ammonia acetate in IPA:Methanol (4:6 v/v) +0.05% Acetic acid. The column flow rate was 0.3mL/min. The autosampler and UPLC column chamber were set at 4°C, 50°C, respectively. The injection volume was 1 μL. The calibration curves in 1 – 5000 ng/mL range were built to quantify plasma free fatty acids. Peaks were evaluated by the Mass Hunter 12.1 software (Agilent Inc., Santa Clara, CA, USA).
