# Supplementary material for: Oral Multienzyme Supplementation Alters Postprandial Plasma Nutrient Concentrations after a Mixed Meal in Healthy Middle-Aged and Older Adults: A Randomized, Double-Blind, Placebo-Controlled, Crossover Trial
Source: J Nutr. 2026 Feb 7;156(4):101400. doi: 10.1016/j.tjnut.2026.101400 (PMC13084569; doi:10.1016/j.tjnut.2026.101400)
Supplement: Multimedia component 2 [file mmc2.pdf]

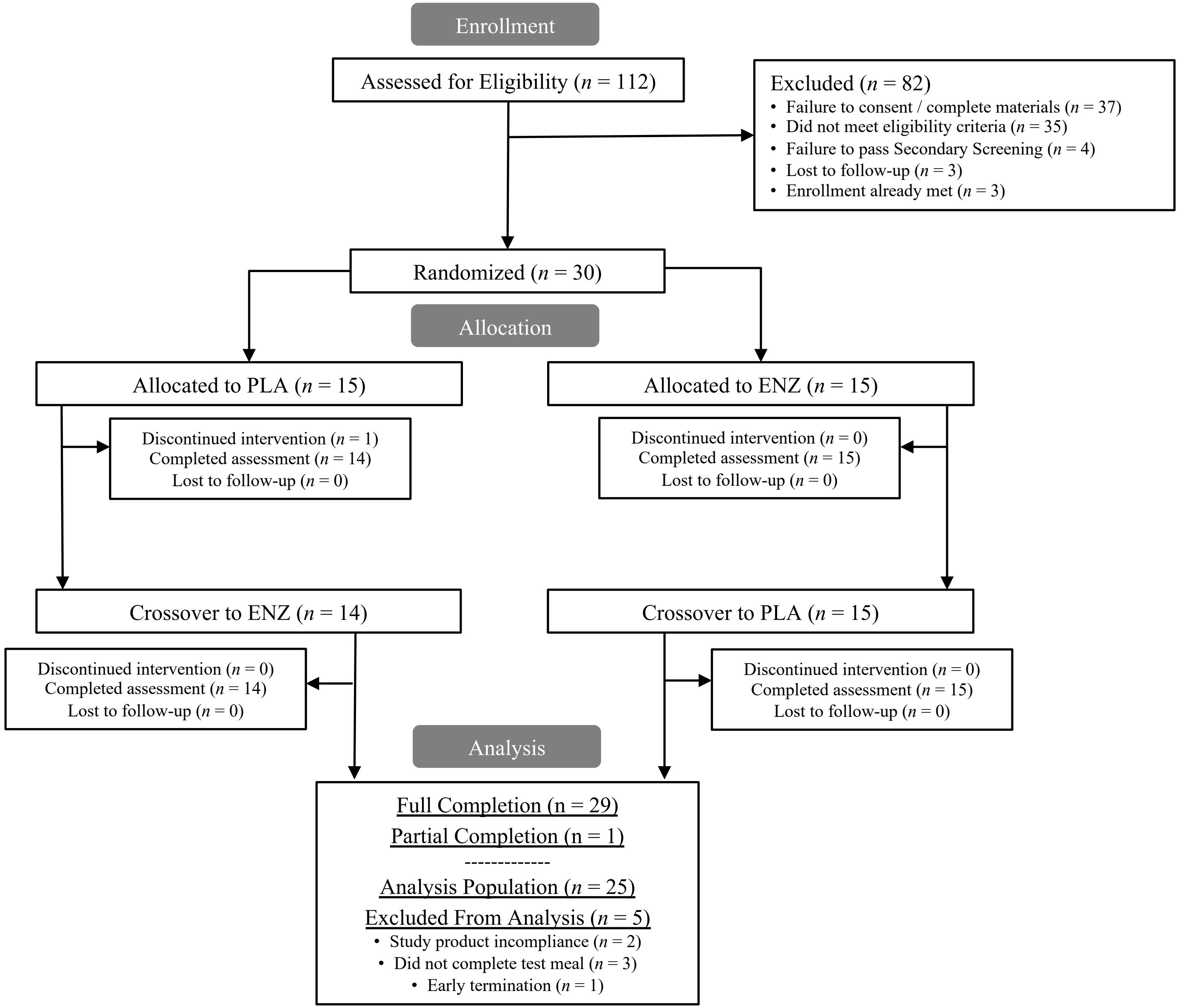

Figure. Participant flowchart. (Abbreviations: PLA = placebo; ENZ = mixture of six microbial enzyme preparations)
